# Observations on the Effects of Irritation upon the Deposition and Absorption of Dental Bone

**Published:** 1847-10

**Authors:** W. H. Elliot


THE AMERICAN

### Journal of Dental Science.

Vol. VIII.] OCTOBER, 1847. [No. 1

### ARTICLE I.

*Observations on the Effects of Irritation upon the Deposition and Absorption of Dental Bone.* Read before the American Society of Dental Surgeons, at their Eighth Annual Meeting, held at Saratoga, August 3, 1847, By W. H. Elliot, D. D. S.

In a paper we had the honor of reading before the Medico-Chirurgical Society of Montreal, on the 3d of April last, we discussed briefly among several subjects, some of the theories which have been offered in explanation of the mysterious phenomenon of the loss of the temporary fang, but as our remarks at that time, embraced several other subjects, we had not an opportunity of considering this one, as fully as we desired; we have, therefore, chosen it for the present occasion.

There is, perhaps, no subject connected with our own branch of the healing art, that has offered a broader field for speculation, or, that has elicited more conflicting opinions, than the shedding of the temporary teeth. We have often had our attention directed to the subject, and our curiosity awakened to know, why teeth of the same class frequently make their appearance earlier upon one side of the mouth, than upon the other, and why a similar but more striking irregularity, occurs in the action of the absorbing vessels. Many writers have invented theories by which they can readily account for the loss of the first set of teeth, under ordinary circumstances, but, inasmuch as they do not account for the exceptions to these, their propositions cannot be received as correct. The irregularity with which this process is carried on, and its apparent dependence upon local causes, renders it more than probable, that the period of its occurrence is not in any way connected with constitutional influences; hence the necessity of fixing upon some theory, which shall explain it satisfactorily, under the various circumstances in which it appears.

In presenting our own views upon this subject, we are aware that we shall differ with men for whose opinions we have ever entertained the highest respect; we shall, therefore, submit them, with some hesitation, yet with a firm conviction of their truth.

Mr. Bell calls the shedding of the temporary teeth a process of anticipation, and believes that it is carried on independently of the necessities of the object for which the change is effected; or, in other words, that it is not the pushing forward of the permanent tooth, that causes the removal of the temporary root, but merely its presence is sufficient to produce it; but at the same time admits, that "it is not until the permanent tooth can no longer be retained in its own alveolus, that the process of absorption commences to open the required space." Now if this process be independent of the permanent tooth, why does it, under ordinary circumstances, always commence at that moment, when more room is required? Why is it carried on in the direction in which the tooth is inclined to grow, and why does it always cease with the growth of the tooth; these facts certainly favor the argument, that absorption is, to a very great degree, dependent upon the advancement of the permanent tooth. We have in our possession, the inferior jaw of an adult, which has the two wisdom teeth fully developed in its structure. These teeth lay horizontally in the jaw, with their grinding surfaces resting against the second molares, and their fangs extending under the *coronoid* processes. Over these crowns, no absorption took place; but, according to Mr. Bell's theory, their presence should have been sufficient to open a passage, whether their natural growth pushed them in that direction or not. Yet this was not the case, nor is it ever the case when the tooth takes a horizontal position, therefore we think it cannot properly be called a process of anticipation.

Mr. Fox tells us that the absorption of the temporary fang, is induced by direct pressure of the permanent tooth upon it; but how can this be reconciled with the fact that dental structure contains no absorbents, and, consequently, when the tooth of replacement, comes in contact with the temporary one, absorption, by the direct action of vessels cannot take place, for the membrane which contained those vessels, is destroyed; for this reason it cannot, as he believes, be the result of direct pressure upon the substance of the fang.

There are cases in which a total destruction of the temporary fang, has taken place, while the tooth of replacement was yet confined to its bony follicle; and Mr. Fox himself, mentions this fact, as a conflicting argument to his own theory, without attempting to answer it. But we shall endeavor to show that absorption of the fangs of one tooth, may be induced by the irritation consequent upon the ulceration of another; and if we admit that the absorbents may be stimulated to action by irritation, we need not look far for an explanation of such cases, whenever we meet them.

The development of teeth in the solid structure of the jaw, affords abundant evidence, that absorption of the bone is not induced by actual contact of the growing tooth. When, by accident, the tooth is prevented from pushing forward through the gum, it remains imbedded in the jaw, and as it increases in length, by a deposition of dental bone from the pulp, the absorbents contained in the sac, or membrane which covers the pulp, carry away the jaw as fast as space is required for the newly formed fang. And so it continues to grow to precisely the same form and size, that it would, under the most favorable circumstances. It is evident that direct contact in this case, would not only prevent the excavation of the jaw, but it would render the growth of the teeth impossible, for the organs which perform these two offices could not exist at all.

Dr. Goddard says, "the shedding of the temporary tooth, depends chiefly upon its death, produced by a loss of arterial supply. When the permanent tooth," says the same author, "impinges upon the end of the fang of its predecessor, it cuts off its supply of arterial blood, thus producing its death." To overturn this theory, we have but to mention a single fact, which is already well known to every one who has any experience in that branch of surgery, upon which, Dr. G. seems so anxious to enlighten us. The vessels of the tooth frequently remain entire, performing their natural functions in the crown, long after the fang has been absorbed, and thus they continue to carry their fluids to the remaining portion of the tooth, while every thing else about them is swept away by the process of absorption. Therefore, it cannot be true, that the destruction of the fang depends upon its death, by the loss of arterial supply.

We could not wish for this subject a more lucid examination, than it has already received at the hands of Dr. Harris. This gentleman's thorough knowledge of anatomy and physiology, has enabled him, in a most accurate manner, to follow out all the changes that occur in the parts concerned, but it is somewhat a matter of regret with us, that he has not offered his opinions on the cause of these changes under the many forms which they appear.

The agency of the carneous substance, discovered by Bourdet, in the removal of the temporary fang, is generally admitted, but that this tubercle is nothing more than an altered condition of the outer membrane of the dental sac, and peduncle, and that this change may take place in any part of this membrane, must also be admitted. The alveolo-dental periosteum of both sets, are also subject to the same change, and it always takes place to a certain degree, where irritation is most severe; the membrane first becomes more vascular, and as soon as absorption commences, it becomes thicker, and the absorbents are undoubtedly most active where this membrane is most vascular.

It has been urged in proof of the agency of this fleshy tubercle in the removal of the temporary fang, that when it "fails to be developed, or is destroyed by an injurious operation, the tooth often remains in its socket, and does not make its appearance;" this may be true, but instead of sustaining the opinion that absorption cannot go on, without the assistance of the tubercle, it completely overturns it; for when the tooth of replacement cannot come forward, absorption must go on, to a still greater extent, so as to make room in the solid structure of the jaw, for the newly formed fang, and this is done without the assistance of the tubercle; therefore, we are bound to believe, that the change necessary to absorption, may take place in any part of the alveolo-dental periosteum, for "it is certainly unphilosophical to attribute a phenomenon to two distinct causes when one alone is sufficient for its explanation."

Some writers have favored the opinion that the vessels of the sac, exhale a dissolving fluid, which decomposes the temporary fang, and thus it is readily carried away by the absorbents, but the fact that we sometimes find portions of the membrane adhering firmly to the eroded surface of the fang, renders the matter doubtful; besides we do not recollect having seen upon the enamel of a tooth of replacement, anything like the effects of a solvent, though cases often occur in which the permanent tooth must have been freely bathed in that fluid, if it has existence.

Many other theories have been advanced for the explanation of the peculiar process, by which nature removes the first set of teeth, but none of them furnish that evidence of truth, which a correct theory should do. They do not account for exceptions as well as general rules.

For the purpose of illustrating our own views on this subject, we shall mention one out of many cases which have come under our notice.

During the fall of 1844, Mrs. G. called on us with her little daughter, who was suffering from alveolar abscess over the root of the right superior central incisor of the temporary set. The crown of this tooth was entirely destroyed by caries, and the root was rendered so sensitive by the existing inflammation, that we were unable to effect its removal in its present state, we, therefore, scarified the gum, and sent her away with directions to call again when the inflammation had subsided. This, however, she neglected to do for more than a year, during which time she had several attacks of periosteal inflammation; and when she did call, absorption of the gum had taken place to such an extent, that the fang was exposed its whole length, we removed it, and on finding the left central incisor loose, we removed that also, and to our surprise, we found that the root of this tooth was entirely absorbed, while the root of the diseased one was whole. In a few weeks after, the right central incisor of replacement cut the gum, and when we last saw our little patient, the crown of this tooth was entirely through, beautifully and perfectly formed; but the eruption of the left permanent incisor had not then taken place.

Now if the case of which we have just given a brief history, were an unusual one, it would mean nothing, but as all the circumstances connected with it are of common occurrence, we may safely draw from it the following inferences:

1st. That an increased but healthy deposit of dental structure, may be produced by local irritation.

2d. That absorption of dental structure may be induced by local irritation.

And, lastly—If the absorbents become too deeply involved in the disease, their operations will be entirely interrupted, and the root, which it was their office to remove, will remain whole.

Premising that the innate constitution be good, and that the blood bear a proper proportion of the earthy salts and gelatine, may it not be fair to suppose that if a larger amount of these materials be brought to the secretory organs, through the circulation, that they, in obedience to this impulse, will be stimulated to greater activity, and thereby advance the natural growth of the tooth.

The absorbents, when active, are always enlarged, upon this ground may we not make the deduction fairly, that they are always active when enlarged by the stimulating influence of local irritation, or, in other words, when enlarged by healthy action. If so, those circumstances in the case mentioned, which gave rise to our second conclusion, are explained.

Cases exhibiting the effects of irritation upon the absorbents, are met with in almost every young patient, so that those who desire an opportunity of testing our theory, need not wait long for it. The fact, that the temporary fang is sometimes absorbed before the carneous substance has reached it, is evidence that its own periosteum must contain absorbents capable of removing it; then we have only to provide these vessels with a stimulant, and they will at once attack every thing in contact with them. That a slight degree of irritation will increase their size and render them active, will, we trust, be admitted when a few examinations have been made. In making these inquiries, it should be remembered that the same amount of irritations, owing to certain constitutional tendencies, will not have the same effect upon the absorbents in all cases. That the destruction of a tooth by caries, and its final removal at an early period, though it may have produced considerable irritation, would not exhibit any outward signs of an increased deposit of dental bone upon the permanent pulp; and the filling up of the temporary alveolus, would retard the progress of the tooth of replacement, more than enough to counterbalance the influence of any previous inflammation. Ulceration of a temporary tooth might destroy the peduncle, and a portion of the sac of its successor, and thereby prevent it from making its appearance at all. But these, and many other circumstances, which may seem to furnish arguments against our conclusions, may readily be explained.

In consideration of the various phenomena of absorption in the buccal cavity, we are induced to believe that its immediate causes, in young subjects particularly, are entirely local, and in no way connected with constitutional influences. Admitting that irritation, or inflammation, in one portion of the jaw may stimulate a greater degree of activity, the absorbents in a neighboring part, and we at once remove the stumbling-block over which nearly all former theories have fallen.

In the shedding of the temporary teeth, absorption is always the result of irritation produced as a general thing, by the pushing forward of the tooth of replacement, though we have abundant evidence that it may be produced by causes more remote. In alveolar abscess, the absorption of this process is the effect of irritation resulting from a decomposition of the nervous pulp. In regulating the teeth of children, the absorption of the alveolus is produced by pressure upon its periosteum. The irritation arising from salivary calculus, badly constructed artificial teeth, &c. frequently cause absorption of the gums and jaw to such an extent that the teeth fall out; and in all these cases the appearance of the alveolo-dental periosteum is the same, that is, it becomes thickened and more vascular.

In conclusion, we have only to remark, that we have found it much easier to demolish the theories of others, than to render our own invulnerable, and though each new inquiry still confirms our belief, yet in submitting it to the examination of a tribunal from whose decision there is no appeal, and to whom so many writers have looked in vain for a confirmation of their opinions on this subject, we are prepared to see it set aside for one more rational; and, whether our exertions result in the establishment of our own theory, or the discovery of the true one, we shall be equally gratified.

